# Existing evidence on the use of participatory scenarios in ecological restoration: a systematic map

**DOI:** 10.1186/s13750-023-00314-1

**Published:** 2023-11-30

**Authors:** Eleanor Durrant, Pete Howson, Bekah Puttick, Samantha Potts, Yara Shennan-Farpón, Novieta Sari, Nicholas Allen, Jo Yeongeun, Matthew Grainger, Yit Arn Teh, Marion Pfeifer

**Affiliations:** 1 School of Natural and Environmental Sciences, Newcastle University, Newcastle Upon Tyne, UK; 2 Department of Geography and Environmental Sciences, Northumbria University, Newcastle Upon Tyne, UK; 3 Norwegian Institute for Nature Research, Torgarden, Postbox 5685, 7485 Trondheim, Norway; 4 Department of Anthropology, University College London, 14 Taviton St, London, WC1H 0BW UK; 5 Geography Department, Bush House (North East Wing), King’s College London, Aldwych, London, WC2B 4BG UK; 6 Department of Communication, Universitas Siber Asia, Jakarta, Indonesia

**Keywords:** Evidence synthesis, Stakeholder engagement, Collaboration, Alternative futures, Socioecological systems, Co-production

## Abstract

**Background and context:**

The scale of land degradation worldwide has led to nearly one billion hectares committed to restoration globally. However, achieving such restoration targets will necessitate complex trade-offs against limited time, competing knowledge, costs, resources and varying stakeholder and societal preferences. Participatory scenarios allow a way to identify collaborative solutions for restoration planning and implementation best suited for the local cultures and societies they are tied to. They can be used to navigate uncertainties surrounding future trajectories of restored areas by evaluating trade-offs in outcomes. This research aims to systematically map the evidence on the use of participatory scenarios in restoration planning. We use the following research question: What evidence exists on the use of participatory scenarios in ecological restoration? This is answered by examining the characteristics of the evidence base, types of study design, types of outcomes, trade-offs in outcomes, and the role of participants.

**Methods:**

A comprehensive and reproducible search strategy was followed using bibliographic databases, web-based searches, and targeted searching. Search results underwent a two-step screening process according to eligibility criteria. Metadata on key areas of interest were extracted from included texts and were narratively synthesised alongside data visualisations to answer the research questions.

**Review findings:**

18,612 records were initially identified, and 106 articles were included in the final map. Most studies were conducted in Europe and North America, focusing on restoring agricultural land or forests. Most texts used mixed methods and explored multiple outcome types, but environmental outcomes were the most assessed. Within environmental outcomes, indicators for ecological function were assessed more frequently than structural or compositional indicators. The most common reason for choosing outcomes and indicators was stakeholder interest. Trade-offs in social, ecological, and economic outcomes were mainly examined across space using mapping techniques, while far fewer studies looked at trade-offs across stakeholders and time. Participants were mostly included in the scenario creation step and were usually chosen purposefully by the research team.

**Conclusions:**

It is difficult to understand how useful scenarios are for restoration planning because few texts reported how scenarios fed into the process. Despite this, the range of outcomes used and different method types adopted suggests participatory scenarios allow for integrating different knowledge and approaches, alongside facilitating the use of qualitative or semi-quantitative data when this is more appropriate or quantitative data is not widely available. To better use participatory scenarios as a tool for ecological restoration planning, decision-makers can push for greater levels and definitions of participation from the offset of restoration projects with specified, regular, and structured communication and participation channels. We also recommend more systematic methods of participant selection, such as stakeholder analysis. Further research is needed to understand the effectiveness of participatory scenarios in restoration planning and whether the participation of stakeholders was successful in meeting objectives. To improve the evidence base, future studies should clearly evaluate their effectiveness in the restoration planning process and their success in meeting their participatory objectives.

**Supplementary Information:**

The online version contains supplementary material available at 10.1186/s13750-023-00314-1.

## Background

20–40% of global land area is estimated to be degraded [1], adversely affecting 3.2 billion people, approximately 40% of the world's population [2]. The scale and urgency of this issue has led the United Nations to declare 2021–2030 the Decade on Ecosystem Restoration, and nearly one billion hectares are committed to restoring degraded ecosystems globally [3]. Ecological restoration, "the process of assisting the recovery of a degraded, damaged, or destroyed ecosystem to reflect values regarded as inherent in the ecosystem and to provide goods and services that people value" [4], aims to reverse the negative impacts of degradation. However, achieving ambitious restoration targets will necessitate complex trade-offs between costs, benefits, and resources, and seeking consensus between varying stakeholder and societal preferences can be difficult, all against the pressures of limited time. Against this background, there is growing evidence of the value of incorporating traditional or local ecological knowledge in restoration projects through participatory means [5, 6]. This is built on the understanding that a holistic and equitable approach to strategic restoration planning that embraces inherent complexities and includes and respects different forms of knowledge and value frameworks to achieve multiple outcomes is needed [5, 7].

### Participatory scenarios

It is now considered that active stakeholder engagement should underpin long-term restoration success [8, 9], and there is evidence that the probability of positive outcomes for biodiversity and livelihoods increases with the extent to which participants can shape decisions [10, 11]. Stakeholder participation is "a process where individuals, groups and organisations choose to take a role in making decisions that affect them" [12]. In this light, scenarios, defined as "representations or storylines of possible futures" [13], provide a pathway for stakeholder participation in restoration planning. Participatory scenarios are a tool to identify collaborative solutions best suited for the local cultures and societies they are tied to [9, 14] while integrating different epistemologies [15], accommodating diversity in stakeholders [16] and giving them a sense of ownership [17]. Decision-makers can use them as a holistic approach to explore future uncertainties in restoration outcomes [18], the impact of interventions on restoration outcomes [19], prioritise resources, and reduce costs [15, 20]. For instance, Palacios-Agundez et al. [14] worked with stakeholders to downscale global scenarios to local ones. Participants suggested management actions towards achieving their desired scenario framed within their local culture and context. They reported learning to see and understand different perspectives and collaborated on proposing feasible management responses [14]. Despite reports of success in participatory methods, there is also evidence of them failing to meet their objectives [21, 22], potentially undermining motivations for using participatory methods. Guidance for implementing participatory scenarios in restoration planning has been developed to help avoid failings [20]. Metzger et al. [20] consulted an international group of scientists and practitioners to conclude that stakeholders should participate in the whole process, from method planning to creating and reviewing scenarios. This way, stakeholders can decide upon and evaluate restoration scenario objectives, outcomes and possible trade-offs [24].

### Outcomes and trade-offs in restoration planning

Restoration objectives are often posited to achieve ecological success alongside improved social and economic outcomes, such as enhanced livelihoods and climate change mitigation [25, 26]. Scenarios and stakeholder engagement are suggested as key tools to analyse potential trade-offs and co-benefits in these various restoration objectives and outcomes across space, time and stakeholders [20, 27, 28]. Their propensity for integrating diverse data collection and analysis [15, 29] alongside facilitating participatory discussion allows for more social and economic dimensions to be captured in a discipline that has traditionally focussed on evaluating environmental components alone [30–32]. In contrast, Bremer et al., [15] quantified ecological and economic outcomes of scenarios alongside qualitative evaluation of cultural outcomes to understand trade-offs between them. The restoration objectives and scenarios were also developed through discussions with the local community and the landowner, who wanted to incorporate multiple values in their decision-making [15]. Moreover, previous research has noted that ecological indicators appeared to focus on attributes that are more easily measured, i.e., the structure or composition of ecological communities or ecosystems, at the expense of evaluating ecosystem function [31].

In creating and analysing scenarios, teams and participating stakeholders can evaluate the inherent trade-offs that will exist and reduce uncertainty around future outcomes of interventions [33], which can be particularly useful for creating a shared understanding across groups that often have diverse knowledge systems and expectations [34, 35]. Regardless of the approach, trade-offs tend to feature heavily in participant discussions, even when not explicitly addressed [23]. One example of such trade-offs has been described by Etienne et al. [34], who documented that when restoring native grassland, sheep farmers' primary objective was to maintain sheep production while conservationists were concerned with the preservation of native biodiversity, objectives that do not always align [34]. Even if objectives are agreed upon, the interventions suggested to achieve them can vastly differ between stakeholders (as in Sisk et al. [24]). Scenarios help navigate these complexities by assessing outcomes and trade-offs in different restoration objectives or management interventions.

Despite calls and guidance for participatory scenarios in restoration planning [13, 20], Acosta et al. [36] found that only 11% of texts were participatory. Further, there is no systematic understanding of the restoration contexts, involved stakeholder groups, methods used for developing participatory scenarios and application of participatory scenarios in exploring diverse sets of objectives, outcomes, and trade-offs. To address this, we map the evidence on the use of participatory scenarios in restoration planning. We examine how research outcomes are explored, how participants are involved, and the trade-offs considered. We inform how participatory scenarios are currently used within ecological restoration planning by systematically collating and mapping the distribution and abundance of evidence on this topic.

### Role of stakeholders

This systematic map is led by a team at Newcastle University. The authors specialise in ecological restoration, using tools from ecological and social sciences across different geographies. The aims were formulated by the initial review team and then sent to five external experts who gave feedback on the topic, knowledge gaps and synonyms for the search string. The experts were purposefully chosen from the review team networks for their diverse geographic focuses and topic areas. Six additional members were added to the review team for the randomised screening and data coding; they also could contribute to the final map and manuscript if they wished. Through publishing with Environmental Evidence, we have adhered to the Collaboration for Environmental Evidence (CEE) review standards and taken advantage of the peer review process for both the protocol and final systematic map to gain valuable feedback. We did an open call for submissions through social media and networks when undertaking the review. We also produced a lay summary of results to distribute through networks for results dissemination.

### Objective of the review

This review aims to systematically map and present the evidence on the use of participatory scenarios in restoration planning. Due to the mixed methods nature of the literature base, we used the SPIDER framework for question formulation [38] (Table 3).

Sample: People participating in scenarios for ecological restoration planning.

Phenomenon of interest: Ecological restoration across any ecosystem type.

Design: Published literature using future scenarios.

Evaluation: Articles that evaluate those scenarios and their outcomes.

Research type: qualitative, quantitative, and mixed methods published literature, including peer-reviewed studies, book chapters, reports, grey literature publications and student theses.

The primary research question was: What evidence exists on the use of participatory scenarios in ecological restoration?

The secondary research questions are:

1. What are the characteristics of the current evidence base—location, scale, design, restoration intervention type?
2. What types of study designs are used for participatory scenarios in restoration planning?
3. What types of outcomes are explored using participatory scenarios?
4. How are trade-offs in outcomes explored in participatory scenarios?
5. What is the role of participants in the scenario process and outcome determination?

## Methods

This systematic map followed the CEE guidelines and standards for evidence synthesis in environmental management [39] and the Reporting Standards for Systematic Evidence Synthesis [40] (Additional file 1), based on published methods [41].

### Deviations from the protocol

Firstly, we altered this review's primary question and title to "What evidence exists on the use of participatory scenarios in ecological restoration?". This was done to depict the aims and systematic mapping method more accurately and has had no implication on sub-research questions and mapping methodology as outlined in the protocol [41].

Additional inclusion and exclusion criteria were added based on the study format (Table 3). Results were excluded if the format did not provide sufficient information for screening, e.g. presentations or conference proceedings, journalism pieces or proposals of work. Books were also excluded because relevant book chapters were found using the search strategy.

There were some deviations during the targeted organisational searches for grey literature due to difficulties with website's search capabilities and the limited resources of the review team to mitigate them. The search string was edited on the Food and Agriculture Organisation (FAO) library because the initial search of 'scenario' listed 4989 hits. The results could not be exported for screening in Rayyan, and it was beyond the capacity of the review team to review all 4989 within the FAO website. Therefore, we modified the search string to 'scenario participatory restoration' for which all results (896) were screened. On the International Tropical Timber Organisation search, search capabilities were low, showing only the first 100 results of 435; therefore, only the first 100 were screened. The World Agroforestry website yielded 1078 results, but we only screened the first 590 after 250 articles were rejected in a row. Since only five grey literature texts were included in the review of 2040 screened, we are confident these modified searches still allowed us to identify a comprehensive set of texts. We also did not perform the forward and backward citation chasing on all publications that passed full-text screening because this was beyond the capacity of the review team.

There were some minor modifications to the data coding sheet to extract additional details from publications, including columns for the lead author name, institutional address and country, publication type, further detail on scales of scenarios, and describing what was done with scenarios after creation, for instance, whether scenarios were made spatially explicit or if qualitative scenarios were then quantified. A column was added on how the study meets the restoration inclusion criteria. Extra categorisations were added to the data coding sheet to ensure consistency between the open coding of texts and to enable improved identification of trends and knowledge gaps. Economic benefits, aesthetic value and recreational value, were added as categories into 'other aspects of the restoration objective'. River and inland waters, and coastal/mangrove/estuary were added as categories into 'land types being restored'. When categorising how stakeholders were selected, 'chosen purposefully by researchers' was added. We added extra categorisations in the outcomes data coding sheet for the outcomes and indicators, methods of outcome and indicators analysis, and further details. 'Land use land cover' was added as a category when categorising outcomes and indicators; this was also further categorised according to the land types listed in the data coding sheet regarding the focal environment, with the addition of 'species habitat'. The 'level of analysis' for outcomes was removed because most studies had insufficient data to record this consistently.

### Search for articles

#### Search terms and strings

Searches were conducted in the bibliographic databases listed in Table 1, accessed through a Newcastle University institutional subscription. Search results were first imported into EndNote and then into Rayyan for de-duplication and screening by reviewers. The following Boolean search string forms the basis of searches (for specific search details of each database see Additional file 2):

**Table 1:** Bibliographic platforms searched including details of each database, date range and results as of 3^rd^ August 2022, all accessed through Newcastle University institutional subscription

Bibliographic platform	Database option selected	Date range selected	Results
Web of science [42]	All databases:	Maximum date range:	4060
	Web of Science Core Collection	1970–present	
	KCI—Korean Journal Database	1980–present	
	MEDLINE	1950–present	
	Russian Science citation index	2005–present	
	SciELO Citation Index	2002–present	
	Zoological record	1962–2007	
SCOPUS [43]	NA	NA	3691
CAB abstracts [44]	NA	NA	907
ProQuest [45, 46]	Natural sciences collection Social sciences collection	1946–present 1914–present	3656
Lens.org [47]	Scholarly works		3427

Scenario: Scenario* OR forecast* OR backcast* OR futur* OR trajector*

AND

Participatory: participat* OR collabor* OR co-product* OR collectiv* OR stakehold* OR engag*

AND

Ecological: ecolog* OR environment* OR ecosystem*

AND

Restoration: restor* OR reveg* OR regener* OR reforest* OR afforest* OR remediat* OR rehabilitat* OR rewild* OR re-wild* OR "conservation translocat*"

The * character is a wild card and will include words containing any characters on the end of the word, so long as the beginning of the word returns a match. For example, participat* may include participation, participatory, participative.

The search string was developed using Web of Science to test combinations of search terms against a benchmark list of eight articles to ensure comprehensiveness (Additional file 2). All benchmark articles except one not present in the Web of Science bibliographic database were found using the final search strategy. The final search string chosen had a high sensitivity but low specificity (Additional file 3). All date ranges imposed on searches were the maximum allowable per database (Table 1). All searches were done in English with no language limitations, so results with bibliographic data translated into English before indexing would be found. However, searching in English only is a limitation of the searches, alongside targeted searching in the grey literature being biased towards international English-speaking organisations due to the knowledge base of the review team. All bibliographic and the web-based search strings have been uploaded to searchRxiv [48].

#### Web-based search

The web-based search was through Google Scholar, where the first 500 results were screened for a modified search string: "scenario" AND "participatory" OR "collaborative" AND "restoration" OR "regeneration" OR "reforestation" AND "ecological" OR "ecosystem". This is due to the limited search capabilities of the internet search engine.

#### Specialist search for grey literature

Searches for grey literature were done across 17 organisational websites within the ecological restoration field (Table 2). Due to limited searching capabilities, only the term' scenario' was searched for, and all results were screened. The exception was the FAO website where the search string was modified to 'scenario participatory restoration' and all results screened. For the International Tropical Timber Organisation, only the first 100 results were screened due to poor search capabilities, and only the first 590 results on World Agroforestry due to 250 publications being rejected in a row.

**Table 2:** Organisational websites used for specialist search of grey literature including the link, search details and results as of 3rd August 2022

Organisation	Link as of 03/08/2022	Search details	Results
International Union for Conservation of Nature (IUCN)	https://portals.iucn.org/library/	Publications	14
Food and Agriculture Organisation (FAO)	https://www.fao.org/publications/search/en/		896
Society for Ecological Restoration (SER)	https://www.ser-rrc.org/resource-database/	Keyword search	64
Global landscapes Forum (GLF)	https://www.globallandscapesforum.org/#		16
Landscapes for People, Food and Nature	http://peoplefoodandnature.org/	Publications only	5
World Resources Institute	https://www.wri.org/resources	Research	95
Stockholm Resilience Centre	https://www.stockholmresilience.org/	Publications	137
UN-REDD	https://www.un-redd.org/document-library		0
WWF	https://wwf.panda.org/discover/knowledge_hub/		13
Tropenbos International	https://www.tropenbos.org/resources/publications		21
Ecoagriculture Partners	https://ecoagriculture.org/resources/publications/		7
International Tropical Timber Organisation	https://www.itto.int/		100 screened of 435
World Agroforestry (ICRAF)	https://www.worldagroforestry.org/publications-all	Documents and publications	590 screened of 1078
Center for International Forestry Research (CIFOR)	https://www.cifor.org/knowledge/publications/		6
Consultative Group on International Agricultural Research (CGIAR)	https://www.cgiar.org/research/publications/		60
European Forest Institute	https://efi.int/		10
Rainforest Alliance	https://www.rainforest-alliance.org/resource/latest/		6

#### Targeted literature searching and other searches

The bibliographies of four relevant evidence syntheses or publications were screened for relevant literature [20, 23, 36, 49] (n = 255). For the systematic review of Quintero-Uribe [49], the included articles and citing articles were also screened (n = 71). We contacted the lead author of the Acosta et al. [36] review for their list of included articles in their systematic map but received no response.

Social media channels and email lists were used to request submissions of relevant literature (scientific and grey). Targeted calls were placed within the International Union for Conservation of Nature, Science for Nature and People network and the Global Landscapes Forum. All submitted publications (n = 5) were screened for eligibility.

### Article screening and study eligibility criteria

#### Screening

Articles had a two-stage screening process: title and abstract and then full text. Title and abstract screening was conducted using Rayyan [50] according to the eligibility criteria in Table 3. Some grey literature had no abstract and so underwent title screening only. Any articles a reviewer was unsure of were put through to full-text screening.

**Table 3:** Description of each question component using the SPIDER framework [38] and the accompanying inclusion and exclusion criteria for publication screening

SPIDER framework	Question component	Criteria
Sample	Participants	*Inclusion*: The research has some form of participation with stakeholders as defined "A process where individuals, groups and organisations choose to take an active role in making decisions that affect them" [12]. Participation can be at any stage in the scenario construction process, for example, input into scenarios that are then used in modelling, data collection with participants or feedback from participants on scenario outputs Stakeholders include everyone directly or indirectly affected by the restoration planning or future scenarios discussed, but they must be outside the investigation team
Phenomenon of interest	Ecological restoration	*Inclusion*: The publication must address any form of ecological restoration as per the definition "Ecological restoration is the process of assisting the recovery of a degraded, damaged, or destroyed ecosystem to reflect values regarded as inherent in the ecosystem and to provide goods and services that people value" [4] Types of restoration may include, but are not limited to: landscape, species, ecosystem, ecosystem service, native species, invasive species removal, habitat, water catchment, coastal, marine Ecological restoration may be addressed through either of the following criteria: 1. The main goal of scenario building is explicitly for ecological restoration 2. The main goal of the scenario building is not explicitly stated as restoration within the publication however it must be identifiable to coders. For instance, if the study area or associated ecological functions are described as degraded and the scenarios are addressing the future of these components 3. Ecological restoration is not the main aim of building scenarios but features as a possibility from at least one scenario. For example, one scenario may be ecological restoration while another may be conversion to an alternative land use
Design	Future scenarios	*Inclusion*: Publication must build or evaluate as least one 'scenario' per the definition "Plausible representations of possible futures for one or more components of a system, or as alternative policy or management options intended to alter the future state of these components" [9]
Evaluation	Outcomes	*Inclusion*: The systematic map will be displaying what and how outcomes are explored within eligible study types. All types of outcomes are eligible, but they must be explored in a future scenario
Research type/Study design	Qualitative, quantitative, mixed method	*Inclusion*: Methods used may be qualitative, quantitative or a mixed methods approach
Additional criteria	Format of the result	*Exclusion*: Results will be excluded if the format 1. does not provide sufficient information, I.e. there are no associated publication with result, faulty links, presentations, conference proceedings 2. is journalism, i.e. news and magazine articles, 3. is a proposal, i.e. manuals and grant proposals 4. is that of a book because book chapters will be found in the search strategy *Inclusion:* Book chapters, reports, grey literature publications, scientific publications, student theses (BSc, MSc, PhD)

At both screening stages, the primary reviewer screened all articles, while three other reviewers each screened a random 30% subset. The Kappa coefficient was calculated to test for reviewer consistency at both stages [51, 52]. Generally, agreement tended to be considered fair (kappa > 0.21) at title and abstract screening and substantial (kappa > 0.61) at full-text [52] (See Additional file 2 for full results). Any reviewer disagreements were discussed, and the decision to include or exclude the article was made together. Generally, disagreements tended to be due to a lack of clarity in the text, particularly at the title and abstract stage. The reviewers were not authors of any of the articles retrieved. The core review team screened articles in a non-English language using translated bibliographic information at title and abstract stage. At full-text stage they were screened by a reviewer fluent in that language. This required three extra reviewers to join the team to review French, Spanish, Korean and German texts.

#### Eligibility criteria

Each publication was screened according to the eligibility criteria presented in Table 3 to determine inclusion or exclusion. The eligibility criteria are expressly related to each component of the research question based on the SPIDER framework [38]. All publications must also be available in an online format.

### Data coding strategy

All studies that met the eligibility criteria at full-text screening underwent data coding and extraction according to the codebook (Additional file 4). The codebook contains a mix of pre-defined multiple-choice answers alongside open questions and was pilot-tested by three reviewers on the benchmark list of articles. One other reviewer coded a random 20% of articles. Due to the largely qualitative and open questions in the data codebook, we did not quantitatively test for consistency between reviewers, but instead, we held regular meetings and any differences were discussed, agreed upon by the reviewers and then adjusted. If critical information was missing or unclear, we were to contact the study's lead author [41]; however, this was not deemed necessary for any texts.

The data extraction was based on the themes of the research questions and has the following groupings:

1. Data coding of study characteristics: bibliographic information, study context, restoration context.
2. Scenarios: objectives and methods, outcomes, trade-offs.
3. Participation: participant selection, participatory process.

### Data mapping method

The systematic map database with all included articles, bibliographic information and extracted data is presented in a Microsoft Excel Workbook and csv files (Additional files 4, 5, 6, 7). This can be used to find any studies with respect to the evidence of interest eg. studies conducted in a particular country or focussing on a particular environment type. This database is readily updatable with new studies and includes explanations on how the literature is organised and coded for (Additional file 4).

Narrative synthesis alongside bar charts, histograms, a choropleth map, and heat map were used to map the data according to the five secondary research questions and identify knowledge gaps and clusters. All visualisations were made in R v4.2.1 [53], using the tidyverse package collection and ggplot, ggVenndiagram, and maps packages [54–57]. Based on these results, recommendations are made for future research and restoration practitioners.

## Review findings

**Fig. 1:**
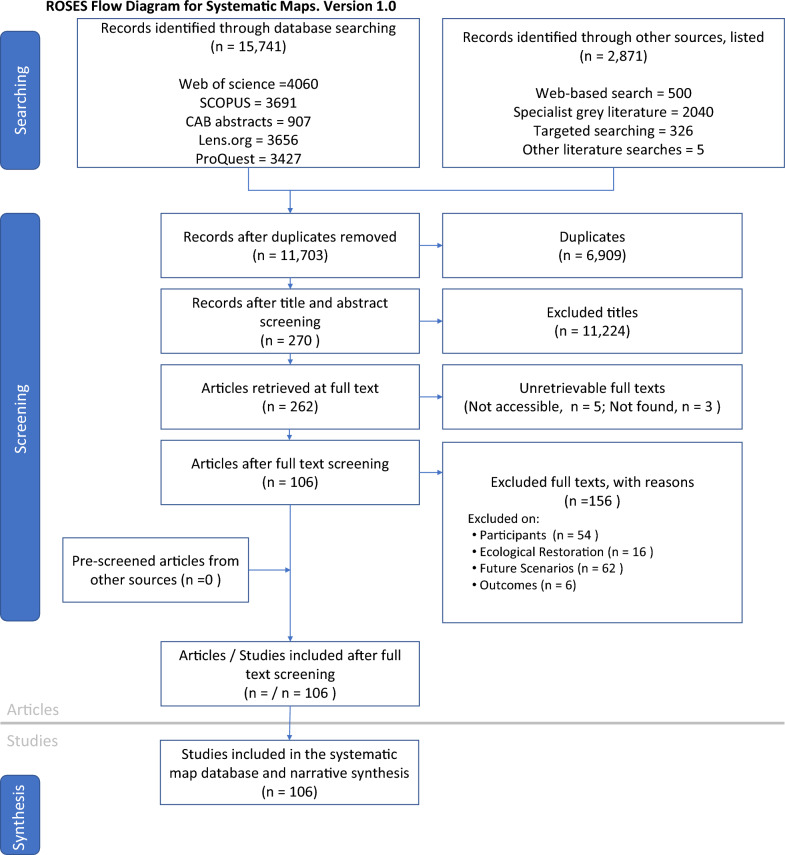
Roses flow chart [58] showing the number of texts at each stage of the review process

Searches were conducted in August 2022. 18,612 records were identified through bibliographic databases (n = 15,741) and other sources (n = 2871) (Fig. 1). After de-duplication, 11,703 unique publications underwent title and abstract screening. 270 articles passed to the full-text screening stage; however, eight records were unretrievable, so 262 were screened: 253 English, two French, three German, one Mandarin, one Korean and two Spanish. 153 texts were excluded at full-stage screening due to not creating future scenarios (n = 62), not being participatory (n = 54), not studying ecological restoration (n = 16) or not examining outcomes of scenarios (n = 6). 106 publications passed full-text screening to be included in the systematic map. Four of these used multiple case studies in which we extracted data for each case study (n = 111). A full list of texts at the full-text screening stage, the reason for exclusion, and which database they were found are available in Additional file 8, including articles that were not found or not accessible.

### Characteristics of the evidence base

#### Publication type and year

**Fig. 2:**
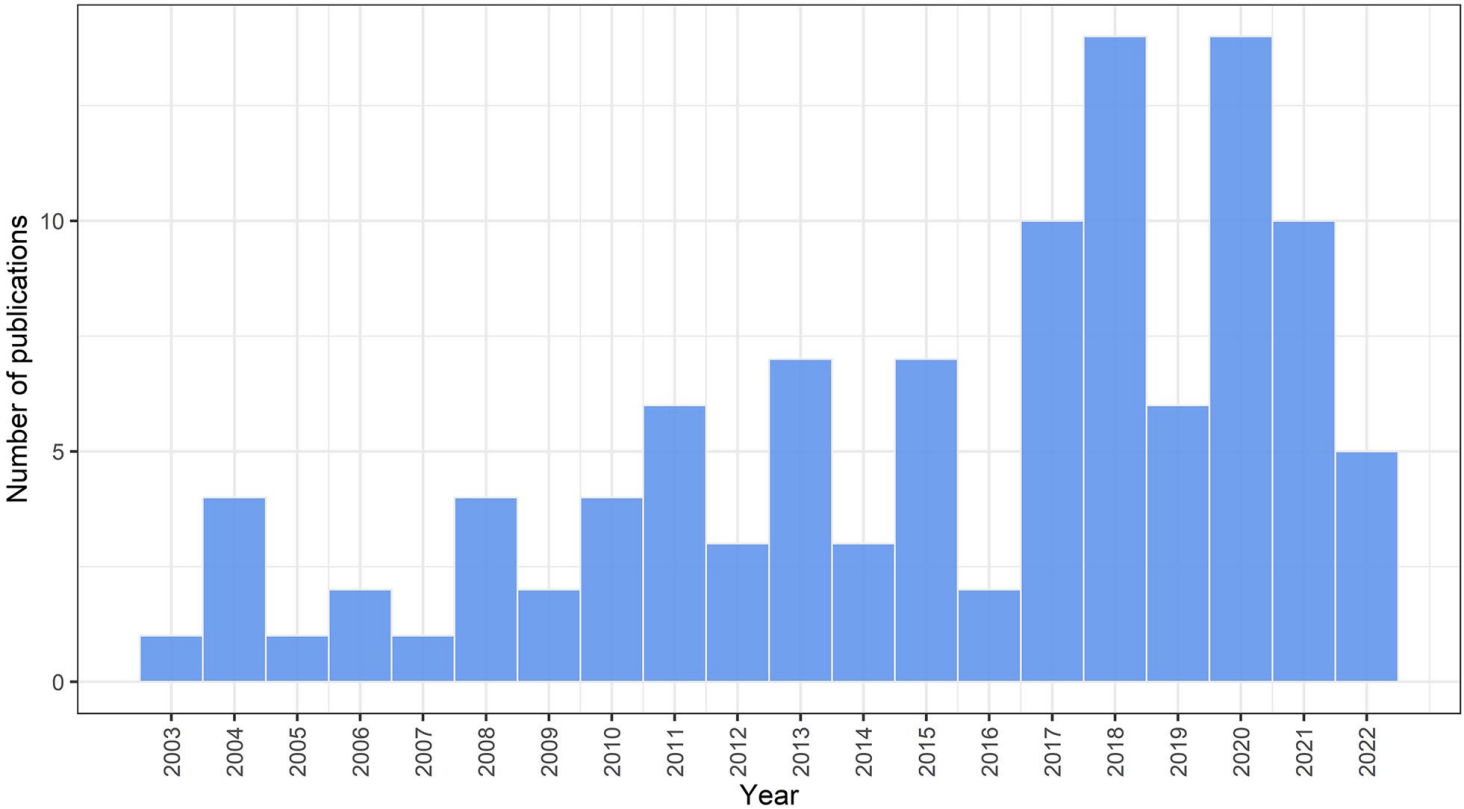
Number of included studies in the systematic map by year of publication

86% of texts were scientific journal articles (n = 91), and 6% were reports. There were three PhD theses, one MSc thesis, three book chapters, and a conference paper. 96 studies had an academic author listed (91%), and 40 studies (38%) listed authors from multiple professions. The earliest text included was published in 2003, with the most studies published in 2018 and 2020 (n = 14) (Fig. 2). 2022 may have a smaller number of studies because the search took place in August of that year and therefore excludes articles published after this time.

#### Study geography and environment

**Fig. 3:**
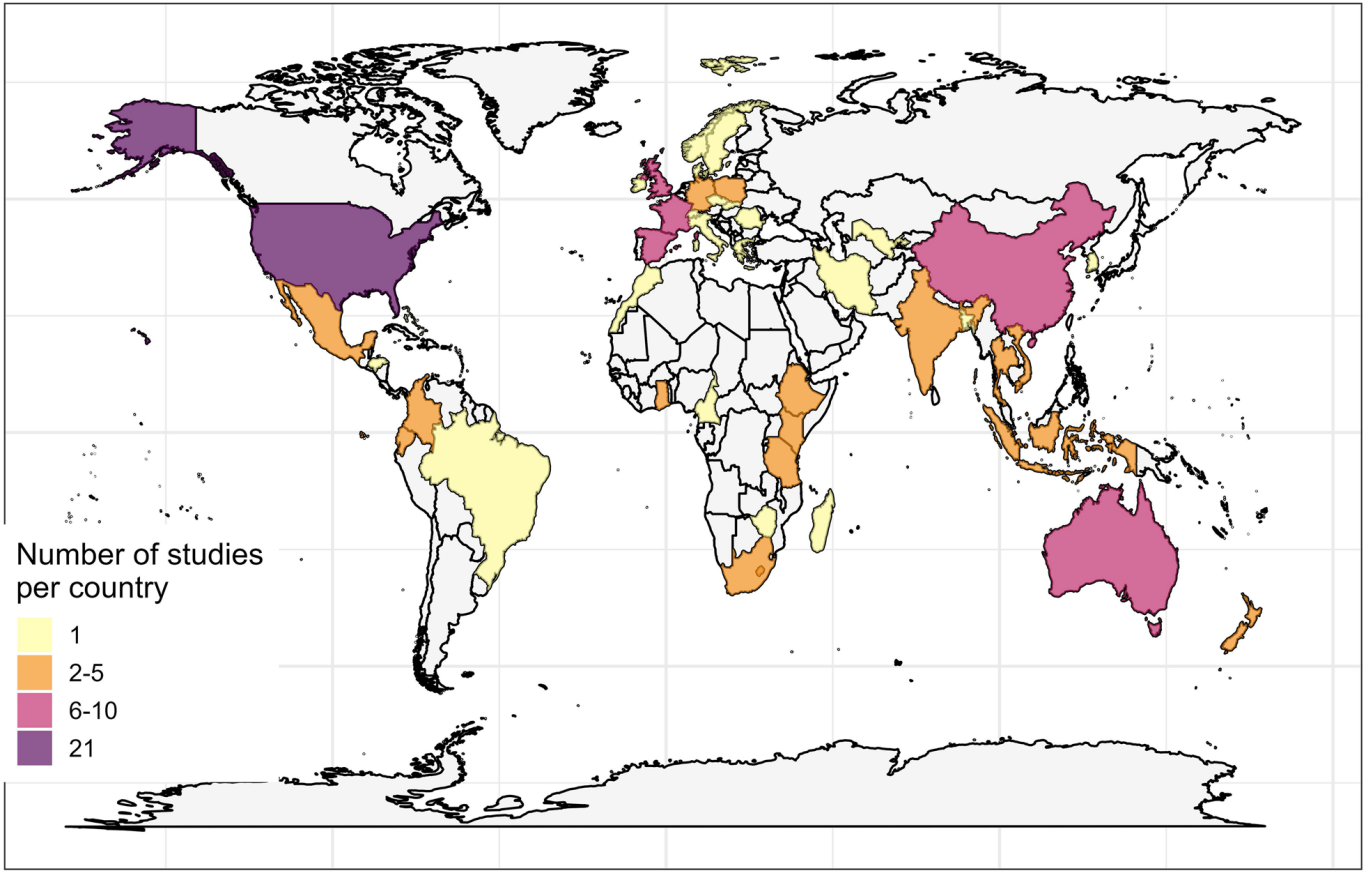
A choropleth map showing the global distribution of studies. Studies spanned 43 countries

Most studies were conducted in Europe (n = 37, 32%), followed by North America (n = 27, 23%), Africa (n = 20, 17%) and Asia (n = 19, 16%). Publications spanned 43 countries, with the most studies conducted in the United States of America (21/116, 20%), followed by France (n = 9/116, 8%) and Spain (n = 8/116, 7%) (Fig. 3). The primary focal environment in most studies were mixed farms and natural vegetation (n = 37, 33%), followed by forest (n = 28, 25%), and coastal and estuarine-related systems e.g. mangroves and sandbanks (n = 15, 14%).

#### Restoration context and scale of scenarios

**Fig. 4:**
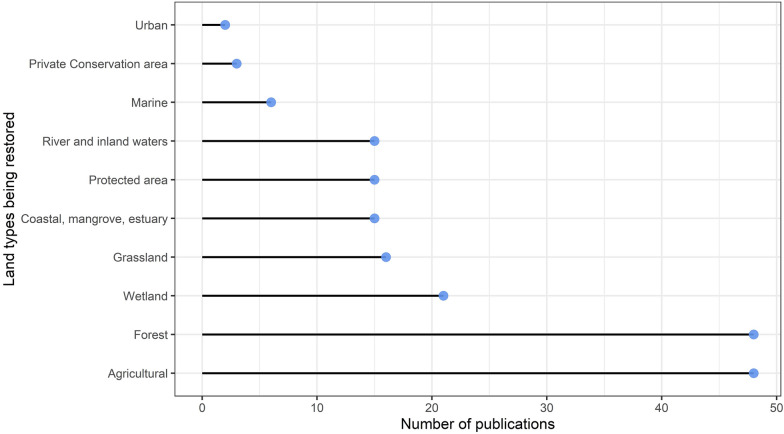
The frequency of land types being restored, some publications examined more than one land type

For 48% (n = 51) of studies, ecological restoration was the primary goal of developing participatory scenarios, whereas in 31% (n = 33), restoration featured but was not the overarching objective of developing scenarios. The remaining studies (21%, n = 18) met the inclusion criteria based on a clear description that the study area was 'degraded', and as such, future scenarios were developed to address the possible alternative states of these degraded components. The dominant driver of degradation was land conversion (n = 55, 23%), followed by urbanisation (n = 33, 14%) and demographics (n = 26, 11%). In the 'other' category, the focus was mainly on the overexploitation of resources and chemical pollution of land and waterways. This relates to land types of focus; most restoration was focused on agricultural land (n = 48) and forests (n = 48) (Fig. 4).

The mean number of restoration objectives listed per text was 3.6, and there is a disparity between the number of ecological objectives (n = 182, 59%) and the number of non-ecological objectives (n = 129, 41%). Habitat restoration was the most popular ecological restoration objective (n = 70, 39%), followed by ecosystem services (n = 61, 34%). Only five studies stated invasive species removal as an objective and only one study to limit disease spread. For non-ecological restoration objectives, economic benefits (n = 19, 15%), livelihood resilience (n = 17, 16%), climate change adaptation and mitigation were the most popular (n = 16, 12%).

Most scenarios were exploratory (exploration of possible futures based on driver trajectories) (n = 52) or policy-screening (forecasting the effects of policy or management interventions) (n = 49) and used a forecasting approach (n = 104) [13]. The spatial scale used for scenarios was usually administrative boundaries (n = 38, 34%), such as regions or districts, or natural features, such as watersheds or floodplains (n = 39, 35%). Only 58% (n = 61) of texts specified a scenario timeframe, of which 20 years was the most popular (n = 11, 18%). Future timeframes ranged from 5 to 96 years, and 4 texts created scenarios over multiple timeframes. The most reported reason for choosing a timeframe was to align with the industry of interest, e.g. Johansson et al. [59] chose a 10-year timeframe that aligned scenarios to agricultural planning and action on the farm level.

53 texts were identified as part of a wider restoration project, but only 28% (n = 30) of texts described how the created scenarios fed back into the project. Usually, scenarios and results contributed to developing a restoration management plan (n = 19, 63%), they were disseminated to decision-makers (n = 4, 13%), or the feasibility of proposed interventions from scenarios was discussed by decision-makers (n = 3, 10%).

### Study designs used in participatory scenarios for restoration planning

Generally, publications were varied in their methods and took a mixed-methods approach (n = 65, 61%). Many built on or combined scenarios with established scientific methods or frameworks such as multi-criteria analysis (n = 10), InVEST ecosystem services valuation models (n = 11), framework for participatory assessment (n = 4), agent-based modelling (n = 3), Bayesian belief networks (n = 4) and fuzzy cognitive mapping (n = 3). This demonstrates the flexibility through which scenarios can be used alongside other methodologies to suit team skills and study contexts.

**Fig. 5:**
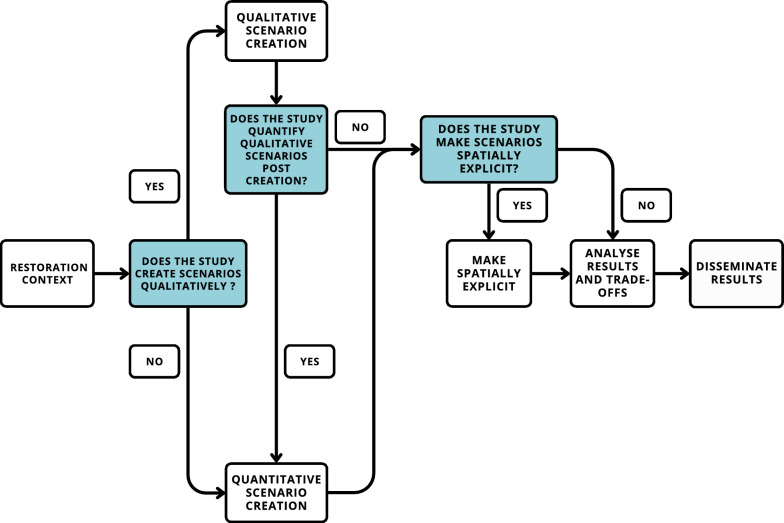
A flow chart depicting popular methodology routes used for scenario creation in restoration planning

When creating scenarios, 43 studies did this qualitatively, and 35 did this quantitatively, with the remainder using semi-quantitative methods (e.g. multi-criteria analysis, n = 16), or a mix (n = 3). Most qualitative studies employed workshops to create scenarios with participants (n = 36, 84%) and 17 studies (40%) used maps as a methodological aid. 14 qualitative scenarios were made spatially explicit during creation, while 26 were made spatially explicit post-creation (Fig. 5). 33 (78%) publications quantified the qualitative scenarios or specific outcomes after creation. Of the quantitative scenarios, 27 were also spatially explicit during creation (77%). Besides modelling techniques, a few also used workshops (n = 11) and group discussions (n = 8) to engage participants when creating scenarios.

### Outcomes and trade-offs explored using participatory scenarios

**Fig. 6:**
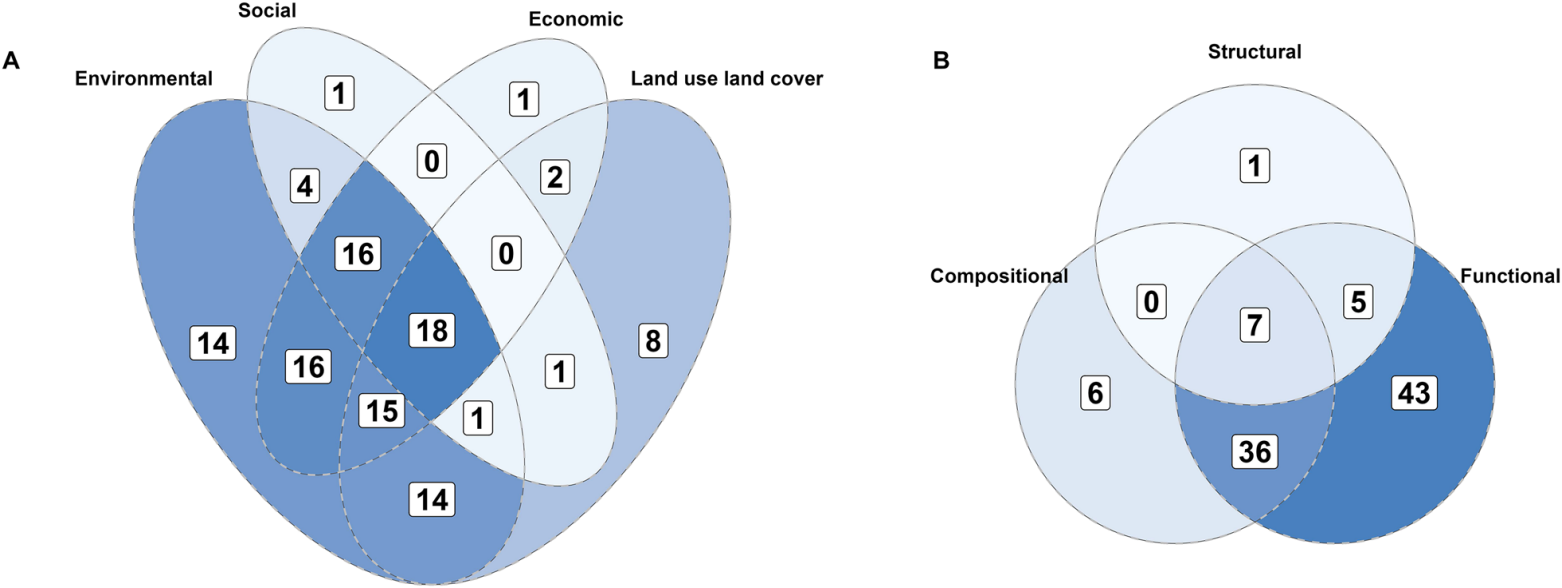
**A** The outcome categories and the combinations of categories explored across publications. **B** The categorisations for environmental outcomes and indicators and the combinations explored across publications

The mean number of outcomes explored was 7, and the median was 5, the lowest number of outcomes studied was 1, while the highest was 59, and many texts had multiple indicators for a single outcome. 88% (n = 98) of texts studied environmental outcomes, followed by economic outcomes (61%, n = 68), land use land cover (53%, n = 59) and social outcomes (37%, n = 41) (Fig. 6A). 78% of studies (n = 86) examined more than one outcome category, demonstrating that scenario planning is useful for integrating outcomes across different dimensions. The most popular described reason for choosing social, ecological, and environmental outcomes and indicators was that they were chosen by or resulted from stakeholder interactions. Whereas for land use land cover, this was to understand better future land use land cover patterns.

Social outcomes tended to be semi-quantitatively or qualitatively examined, and only 11% of social outcomes were quantified compared to 47% of environmental and economic outcomes. Of the 555 environmental indicators, functional indicators were the most studied (n = 385, 69%), followed by compositional (n = 141, 26%) and structural (n = 28, 5%) (Fig. 6B). Under half of the functional (n = 166, 43%) and compositional (n = 58, 41%) indicators examined were quantified. Conversely, structural indicators tended to be quantified (n = 19, 67%).

**Fig. 7:**
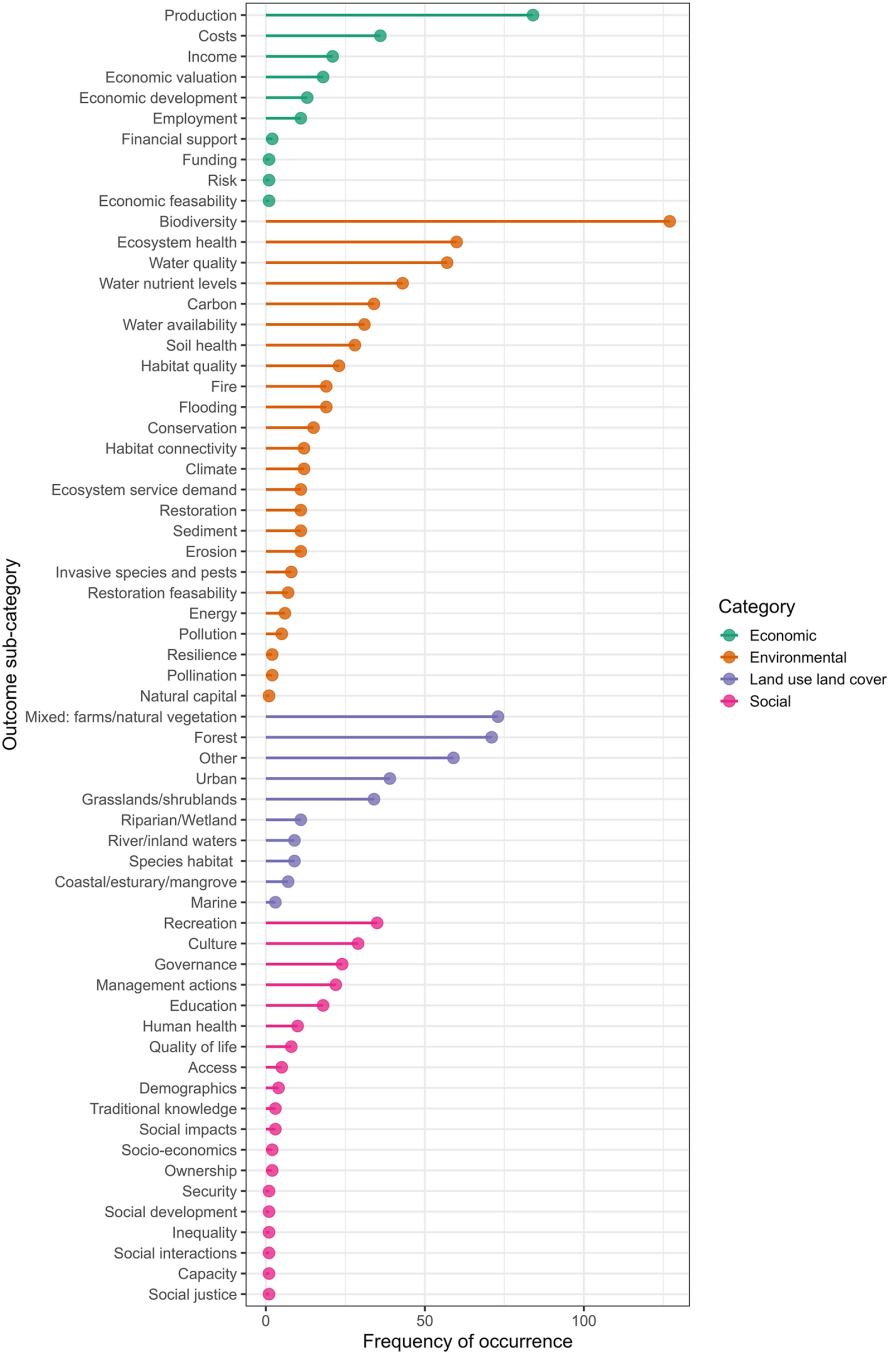
The frequency of indicator sub-categories that were explored across included texts

Within environmental indicator sub-categories, biodiversity was the most evaluated (n = 127, 23%), followed by indicators for ecosystem health (n = 60, 11%), and water quality (n = 57, 10%) (Fig. 7). Economic indicators usually linked to the production of goods such as food and timber (n = 84, 45%), and the costs of scenario interventions (n = 36, 19%). The land use and land cover indicators predominantly focussed on mixed environments of farms and natural vegetation (n = 73, 23%) and forests (n = 71, 22%), reflecting the land use types most commonly being restored. Social indicators primarily related to recreation (n = 35, 20%), culture (n = 29, 16%), and governance (n = 24, 14%).

52 studies explicitly addressed trade-offs between outcomes in the text; however, coders identified a further 47 that addressed them implicitly. Only 9 texts did not address trade-offs in any way. Trade-offs were mainly addressed after scenario construction (n = 79, 75%) and usually within the text (n = 86), using a visual depiction (n = 34), workshops (n = 23) or mathematical modelling (n = 13). 51 publications addressed spatial variation in trade-offs, generally through spatially explicit mapping. 23 texts addressed trade-offs between stakeholders, generally through qualitative discussion, while only 9 publications addressed trade-offs across time by comparing different time steps. 31 adopted a participatory approach when analysing trade-offs, usually through workshop discussion.

### Role of participants in the scenario process and outcome determination

**Fig. 8:**
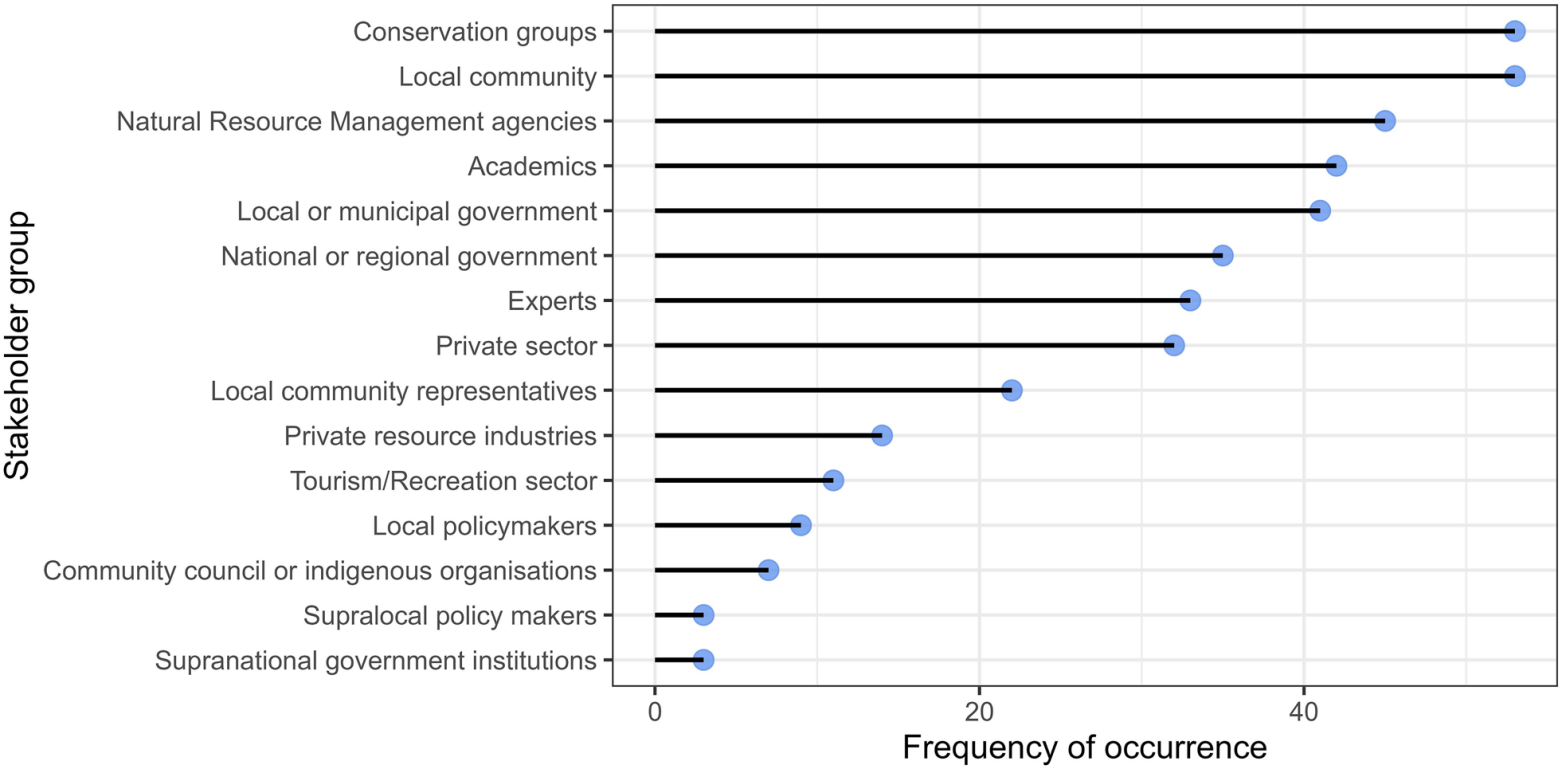
The frequency of different categorised stakeholder groups represented in participatory scenarios across the included texts

The median number of participants was 27, while the minimum was 4 and the maximum was 570. Of those who gave details, most participants were chosen purposefully by the publication team (n = 20). Other common methods of selection include stakeholder analysis, snowball sampling and through a local partner. Members of the local community and conservation groups/non-governmental organisations, co-management groups were the most included stakeholder groups (n = 53) (Fig. 8). Natural Resource Management Agencies, local government and academics were also highly represented. Of the 15 categories for stakeholder groups in the data coding, the mean number of groups was 3.4, and the median was 3.

**Fig. 9:**
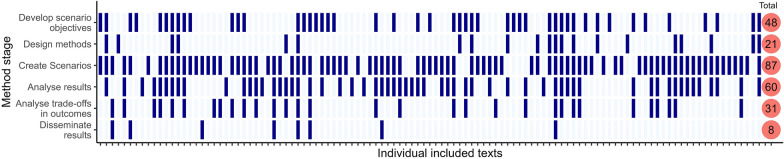
The stages of which stakeholders participated in the scenario process. Blue tiles indicate stakeholder participation, the total number of texts that used participatory methods for each stage is shown in the red circle

We categorised six stages of the scenario process through which stakeholders participated: develop scenario objectives, design methodology, create scenarios, analyse scenarios, analyse trade-offs, and disseminate results. Creating scenarios was the most common stage of participation (n = 83), followed by contributing to the analysis of results (n = 57) and developing objectives (n = 48) (Fig. 9). Very few studies took a participatory approach to results dissemination (n = 8). Only 17 studies specified the duration of stakeholder collaboration and participation, of which 13 explicitly stated collaboration was over years. The only 2 studies that used participatory methods for all stages of the scenario process had multi-year collaborations. Although the length of collaboration is not indicative of participation, some multi-year studies only had one participatory stage. The average number of stages stakeholders participated in was 2. 18 studies had 4 or more stages of participation in the scenario process; these studies tended to have specified, regular, and structured communication and participation channels such as through workshops, meetings, steering groups or working sessions.

Participants were often included in developing scenario objectives and scenario creation through meetings and workshops. Participation in methodological design usually took the form of co-constructing a conceptual or mathematical model and deciding on model formation, inputs, and indicators. For the quantitative scenarios, sometimes participants assisted in parameterising the model (n = 10) or ran the model themselves (n = 4). Participatory scenario analysis and review of trade-offs were often through workshops or follow-up meetings where the study team presented created scenarios to be evaluated and analysed by participants before being revised. The few studies that used participatory methods to disseminate results used workshops to disseminate to other community members, partook in producing media outputs, or influenced dissemination methods.

There was a plethora of reasons for publications using a participatory approach in scenario design, such as to integrate different knowledge types, facilitate a common understanding, accommodate diversity in stakeholders and perceptions, enhance collaboration and a sense of ownership, ensure local relevancy, and increase the likelihood of study acceptance and success.

### Limitations of the map

A limitation of the map was that searches were conducted in English, and only translated bibliographic information would appear in the results. This may have biased article distribution towards English-speaking countries or organisations that publish in English. The high number of articles from French and Spanish-speaking countries indicates that including these languages may have increased the range of the map, but this would still lead to the same bias against countries speaking other languages and was beyond the capacity of the review team. To try and counter these biases, we chose the search strings or terms with high sensitivity but low specificity. Additionally, eight articles were not accessible, or we could not find the full text, and due to the limited capacity of the review team, we had to review and consequently forgo our original strategy of performing forward and backward citation chasing.

Despite pilot testing the coding strategy beforehand, we had to modify the coding sheet to include further details or categorisations. Adding further categorisations allowed consistency between open answers for grouping and comparisons of studies. During data coding, the categorisation of outcomes and indicators into social, ecological, and environmental indicators was sometimes difficult due to the simplicity of the categorisation. For example, the indicator' tuna occurrence' was categorised as an economic indicator because it was under the outcome 'Satisfactory harvest of mahinga kai' (Māori, resources that are customarily used/harvested), and therefore linked more to the productive harvestable economic value of tuna [60]. However, this indicator overlapped with the environmental category because it relates to a species population and the social category, recognising the cultural significance of mahinga kai for Māori people. For these, the reviewers discussed and decided on the best fit, but we recognise that there is a level of value judgment in this. We strived to have a balance between categorising variables to identify trends and knowledge gaps while maintaining the integrity of the data.

## Conclusions

This systematic map explored the use of participatory scenarios in restoration planning, focussing on how outcomes are evaluated and the role of participants. The section below relays the implication of our results for policy/management and research.

### Implication for policy/management

It is difficult to understand how useful scenarios are for restoration planning because few texts reported how scenarios fed into the process. Of those that did, it was common that they supported management plans or were being used by decision-makers. Despite this, our systematic map has shown the flexibility of using participatory scenarios for restoration planning; evidence was distributed across 43 countries and many different types of environments. Study authors were often from different disciplinary and professional backgrounds, which was evident in the wide range of approaches taken, with most studies using mixed-methodologies. Regardless of greater attention to environmental outcomes, they were usually explored across multiple categories (social, environmental, economic, and land use land cover). These were typically included due to the interest of the multiple stakeholder groups involved. This highlights that participants value various restoration outcomes such as water quality, sustainable production, recreation, and cultural values. We found a wide range of outcomes used and method types adopted in the different studies. We suggest that this evidences that participatory scenarios allow for integrating different knowledge and method types, alongside facilitating qualitative or semi-quantitative data when this is more appropriate or quantitative data is not widely available. For instance, Arias-Hidalgo et al. [61] quantified water quality and quantity indicators alongside conducting a multi-criteria analysis with experts for outcome indicators where quantitative information was unavailable, such as touristic potential and biodiversity. This flexibility is advantageous to ensure scenarios are adaptable to stakeholder needs, resources available and the objectives and outcomes being explored.

92% of texts addressed trade-offs in outcomes, supporting that scenarios are suitable for evaluating trade-offs across different methods such as workshops, mapping, or mathematical modelling. Spatial variation in trade-offs was addressed by 52% of texts that examined trade-offs; this was fewer than the review team expected given the spatial nature of ecological restoration interventions, but may be reflective of the high number of exploratory scenarios created.

Regardless of guidance to include participants in all stages of the scenario process for restoration planning [20], only 2 texts did this, and very few studies took a participatory approach to method design and results dissemination. Moreover, only 31% of the texts analysed trade-offs in a participatory manner. To better use participatory scenarios as a tool for ecological restoration planning, decision-makers can push for greater participation from the offset of restoration projects, particularly in the stages before and after scenario creation, as recommended elsewhere [20]. Despite a wide range of methods, texts that included participants in more stages of the scenario process tended to have specified, regular and structured communication and participation channels. There was a high representation of stakeholder groups interested in ecological restoration, such as conservation groups and natural resource management agencies. Communication intensity and participants' environmental stance have recently been shown to improve environmental governance outcomes [11]. Participants were most often chosen purposefully by the study team and despite advantages such as the ability to select the most beneficial participants, this method can also be vulnerable to researcher bias and an inability to generalise [62]. Systematic methods such as stakeholder analysis are recommended in the literature to ensure the whole range of perspectives and interests are represented [20, 63].

### Implication for research

Now that there is a systematic map of how participatory scenarios are being used, further research is needed to understand the effectiveness of participatory scenarios in restoration planning. Due to the wide range of the publication base and lack of this information within publications, this would need to be done by conducting primary research into the included studies in this review with study teams and the restoration decision-makers.

This is also the case in understanding whether stakeholders' participation succeeded in meeting the objectives for undertaking a participatory approach. Newig et al. [11] found that the extent to which participants can shape decisions was the strongest predictor of positive outcomes for environmental governance. Stakeholders on average, only participated in 2 stages of the participatory scenario process across studies. Further analysis of included texts using their framework may better indicate how participatory studies are and how likely they are to achieve positive outcomes for restoration planning. To improve the evidence base, future participatory scenarios should evaluate their effectiveness for scenarios in the restoration planning process and their success in meeting their participatory objectives.

## Supplementary Information

**Additional file 1.** Roses checklist.

**Additional file 2.** Search strategy development including search string by database, test list of benchmark articles and the kappa coefficient in screening.

**Additional file 3.** Search string building details.

**Additional file 4.** Codebook metadata excel file includes all metadata extracted and descriptions.

**Additional file 5.** Condensed human-readable file of main metadata frame.

**Additional file 6.** Metadata frame of participatory scenario outcomes.

**Additional file 7.** Machine-readable file of main metadata frame.

**Additional file 8.** List of texts at full-text screening stage including articles that were excluded, not found or not accessible.

## Data Availability

All data generated or analysed during this study are included in this published article [and its supplementary information files].
