# Supplementary material for: Existing evidence on the use of participatory scenarios in ecological restoration: a systematic map
Source: Environ Evid. 2023 Nov 30;12:27. doi: 10.1186/s13750-023-00314-1 (PMC11378857; doi:10.1186/s13750-023-00314-1)
Supplement: Supplementary file 1 — Additional file 1. Roses checklist. [file 13750_2023_314_MOESM1_ESM.pdf]

Item number	Section/sub-section	Topic	Description	Further explanation	Checklist/meta-data	Author response	Comments
1	Title	Title	it is an update/amendment: e.g. "...A systematic map update."	question.	Meta-data	The role of participatory scenarios in ecological restoration: a sys	
2	Type of review	Type of review	map update, systematic map amendment	updates [2]	Meta-data	systematic map	
3	Authors' contacts	Authors' contacts	authors must be provided.		Checklist	Yes	
4	Abstract	Structured summary	structured into separate sections: Background, the context and purpose		Checklist	No	
5	Background	Background	known. Reviews must indicate why this study was necessary and what it	links the intervention or exposure to the outcome.	Checklist	Yes	
6	Stakeholder engagement	Stakeholder engagement	the formulation of the question) must be described and explained (using		Checklist	Yes	
7	Objective of the review	Objective	applicable).	questions are usually linked to sources of heterogeneity (effect	Checklist	Yes	
8		components	intervention(s)/exposure(s), comparator(s), and outcome(s).	For other question types see [4,5]	Meta-data	Yes	SPIDER framework used
9	Methods	Protocol	Provide citation, DOI or open-access link to published protocol.	(open access).	Meta-data	Yes	
10		Deviations from protocol	those set out in the protocol along with a justification.		Checklist	Yes	
11	Searches	Search strategy	dates of searching, institutional subscriptions (or date ranges subscribed		Checklist	Yes	
12		Search string	the string is formatted (e.g. Web of Science format)		Meta-data	reveg* OR	Web of Science format
13		databases	List languages used in bibliographic database searches		Meta-data	Yes, English	
14		Languages – grey literature	search engines		Meta-data	Yes, English	
15		Bibliographic databases	Provide the number of bibliographic databases searched		Meta-data	5	
16		Web-based search engines	Provide the number of web-based search engines searched		Meta-data	1	
17		Organisational websites	Provide the number of organisational websites searched		Meta-data	17	
18		of the search	strategy was assessed (i.e. list of benchmark articles)		Checklist	Yes	
19		Search update	review	original searches were performed more than two years prior to review	Checklist	n/a	
20	inclusion criteria	Screening strategy	Methods for consistency of screening decisions (at title, abstract, and full		Checklist	Yes	
21		Inclusion criteria	articles/studies. These must be broken down into the question key		Checklist	Yes	
22	Critical appraisal	Critical appraisal strategy	(including assessment of individual studies and the evidence base as a	Optional	Checklist	n/a	
23		synthesis	synthesis.	Compulsory if critical appraisal performed	Checklist	n/a	
24	strategy	strategy	providing lists of variables that will be extracted as meta-data and those		Checklist	No	
25		Approaches to missing data	information or data from authors.		Checklist	Yes	
26	Data synthesis and presentation	Narrative synthesis strategy	the form of descriptive statistics, tables (including SM database) and		Checklist	Yes	
27		identification strategy	gaps (unrepresented or underrepresented subtopics that warrant further		Checklist	Yes	
28		independence	articles to be considered within the review) in decisions regarding	review should be prevented from unduly influencing inclusion decisions,	Checklist	Yes	
29	Results (review findings)	Description of review process	from all sources and retained through each stage of the review. Must		Checklist	Yes	
30		Number of search results	(including updates if conducted) prior to duplicate removal.	organisational website searches: this will help assessment of the	Meta-data	15741	
31		duplicate removal	searches following duplicate removal.	organisational website searches: this will help assessment of the	Meta-data	11703	
32		Full text screening excludes	Additional file containing list of and reasons for full text exclusions.		Checklist	Yes	
33		Title screening results	Provide the number of articles retained following title screening.	Optional if screening titles and abstracts together	Meta-data		
34		Abstract screening results	Provide the number of articles retained following abstract screening.	Optional if screening titles and abstracts together	Meta-data		
35		results	screening.	Optional if screening titles and abstracts separately	Meta-data	270	
36		Retrieval results	Provide the number of articles retrieved at full text.		Meta-data	262	
37		Unobtainable articles	Additional file containing list of unobtainable articles.		Checklist	Yes	
38		Full text screening results	Provide the number of articles retained following full text screening.		Meta-data	106	
39		Consistency checking: screening	extraction and coding, critical appraisal) must be provided. Provide the		Checklist	Yes	
40		Narrative synthesis	avoiding vote-counting (tallying of studies based on results; direction or		Checklist	Yes	
41		Systematic map database	Additional file containing meta-data and coding for included studies.		Checklist	Yes	
42		Limitations of the review	Discuss possible limitations in the methods used.		Checklist	Yes	
43		Limitations of the evidence base	Discuss possible limitations in the evidence base.		Checklist	Yes	
44	Conclusions	Knowledge gaps and clusters	that warrant further primary research) and knowledge clusters (well-		Checklist	Yes	
45		policy/management	the identified evidence may inform policy/practice decision making in	recommendations or advocacy.	Checklist	Yes	
46		Implications for research	including options for increasing the reliability of study design that could	is permissible provided it is clearly justified by the review	Checklist	Yes	
47	Declarations	Competing interests	review authors may have.		Checklist	Yes	

References

[1] James, K.L., Randall, N.P. and Haddaway, N.R., 2016. A methodology for systematic mapping in environmental sciences. Environmental Evidence, 5(1), p.7.
[2] Bayliss, H.R., Haddaway, N.R., Eales, J., Frampton, G.K. and James, K.L., 2016. Updating and amending systematic reviews and systematic maps in environmental management. Environmental Evidence, 5(1), p.20.
[3] Haddaway, N.R., Kohl, C., da Silva, N.R., Schiemann, J., Spök, A., Stewart, R., Sweet, J.B. and Wilhelm, R., 2017. A framework for stakeholder engagement during systematic reviews and maps in environmental management. *Environmental Evidence* , *6* (1), p.11.
[4] Collaboration for Environmental Evidence. 2018. Guidelines and Standards for Evidence synthesis in Environmental Management. Version 5.0. www.environmentalevidence.org/information-for-authors.
[5] Leeds Institute of Health Sciences. https://medhealth.leeds.ac.uk/info/639/information_specialists/1500/search_concept_tools. Accessed 12/11/2017.
