# Supplementary material for: Existing evidence on the use of participatory scenarios in ecological restoration: a systematic map
Source: Environ Evid. 2023 Nov 30;12:27. doi: 10.1186/s13750-023-00314-1 (PMC11378857; doi:10.1186/s13750-023-00314-1)
Supplement: Supplementary file 2 — Additional file 2. Search strategy development including search string by database, test list of benchmark articles and the kappa coefficient in screening. [file 13750_2023_314_MOESM2_ESM.docx]

Search strategy development

Table of Contents

[Search string by database 2](#_Toc142041185)

[Web of science: All databases 2](#_Toc142041186)

[SCOPUS 2](#_Toc142041187)

[CAB abstracts 2](#_Toc142041188)

[ProQuest: Natural and social science collections 2](#_Toc142041189)

[Lens.org 3](#_Toc142041190)

[Google scholar 4](#_Toc142041191)

[Test list of benchmark articles 4](#_Toc142041192)

[Bibliographic information of benchmark articles 4](#_Toc142041193)

[Scope of the test list against SPIDER framework 4](#_Toc142041194)

[Pilot screening of the test list against inclusion and exclusion criteria 6](#_Toc142041195)

[Keyword development for the search string 8](#_Toc142041196)

[Screening 9](#_Toc142041197)

[Kappa coefficient for consistency between reviewers 9](#_Toc142041198)

# Search string by database

## Web of science: All databases

#### Command line:

(((TS=(restor* OR reveg* OR regener* OR reforest* OR afforest* OR remediat* OR rehabilitat* OR rewild* OR re-wild* OR "conservation translocat*") AND TS=(participat* OR collabor* OR co-product* OR collectiv* OR stakehold* OR engag*) AND TS=(ecolog* OR environment* OR ecosystem*) AND TS=(Scenario* OR forecast* OR backcast* OR futur* OR trajector*))))

1864-present.

#### Databases include

- Web of Science Core Collection (1970 – present)
- KCI - Korean Journal Database (1980 – present)
- MEDLINE (1950 – present)
- Russian Science citation index (2005 – present)
- SciELO Citation Index (2002 – present)
- Zoological record (1964 – 2007)

## SCOPUS

#### Command line:

TITLE-ABS-KEY((restor* OR reveg* OR regener* OR reforest* OR afforest* OR remediat* OR rehabilitat* OR rewild* OR re-wild* OR "conservation translocat*") AND (participat* OR collabor* OR co-product* OR collectiv* OR stakehold* OR engag*) AND (ecolog* OR environment* OR ecosystem*) AND (Scenario* OR forecast* OR backcast* OR futur* OR trajector*))

## CAB abstracts

#### Command line:

ti(((restor* OR reveg* OR regener* OR reforest* OR afforest* OR remediat* OR rehabilitat* OR rewild* OR re-wild* OR "conservation translocat*") AND (participat* OR collabor* OR co-product* OR collectiv* OR stakehold* OR engag*) AND (ecolog* OR environment* OR ecosystem*) AND (Scenario* OR forecast* OR backcast* OR futur* OR trajector*)) )

OR

ab(((restor* OR reveg* OR regener* OR reforest* OR afforest* OR remediat* OR rehabilitat* OR rewild* OR re-wild* OR "conservation translocat*") AND (participat* OR collabor* OR co-product* OR collectiv* OR stakehold* OR engag*) AND (ecolog* OR environment* OR ecosystem*) AND (Scenario* OR forecast* OR backcast* OR futur* OR trajector*)) )

Note: keyword search is not available on CAB abstracts

## ProQuest: Natural and social science collections

Natural Science collection 1946 – present

Social science premium collection 1914 – present

#### Command line:

ti(((restor* OR reveg* OR regener* OR reforest* OR afforest* OR remediat* OR rehabilitat* OR rewild* OR re-wild* OR "conservation translocat*") AND (participat* OR collabor* OR co-product* OR collectiv* OR stakehold* OR engag*) AND (ecolog* OR environment* OR ecosystem*) AND (Scenario* OR forecast* OR backcast* OR futur* OR trajector*)) )

OR

ab(((restor* OR reveg* OR regener* OR reforest* OR afforest* OR remediat* OR rehabilitat* OR rewild* OR re-wild* OR "conservation translocat*") AND (participat* OR collabor* OR co-product* OR collectiv* OR stakehold* OR engag*) AND (ecolog* OR environment* OR ecosystem*) AND (Scenario* OR forecast* OR backcast* OR futur* OR trajector*)) )

OR

if(((restor* OR reveg* OR regener* OR reforest* OR afforest* OR remediat* OR rehabilitat* OR rewild* OR re-wild* OR "conservation translocat*") AND (participat* OR collabor* OR co-product* OR collectiv* OR stakehold* OR engag*) AND (ecolog* OR environment* OR ecosystem*) AND (Scenario* OR forecast* OR backcast* OR futur* OR trajector*)) )

#### Page options (peer reviewed)

- Peer-reviewed option selected
- All languages selected

#### Page options (grey literature)

- Peer-reviewed option not selected
- All languages selected
- Document types:
  - Book chapter
  - Case study
  - Working Papers
  - Dissertations and Theses
  - Reports
  - Government and Official publications

## Lens.org

Scholarly works search.

#### Command line:

title: (restor* OR reveg* OR regener* OR reforest* OR afforest* OR remediat* OR rehabilitat* OR rewild* OR re-wild* OR "conservation translocat*")AND (participat* OR collabor* OR co-product* OR collectiv* OR stakehold* OR engag*)AND (ecolog* OR environment* OR ecosystem*) AND (Scenario* OR forecast* OR backcast* OR futur* OR trajector*)

OR

abstract: (restor* OR reveg* OR regener* OR reforest* OR afforest* OR remediat* OR rehabilitat* OR rewild* OR re-wild* OR "conservation translocat*")AND (participat* OR collabor* OR co-product* OR collectiv* OR stakehold* OR engag*)AND (ecolog* OR environment* OR ecosystem*) AND (Scenario* OR forecast* OR backcast* OR futur* OR trajector*)

## Google scholar

#### Command line:

(scenario) AND (participatory OR collaborative) AND (restoration OR regeneration OR reforestation) AND (ecological OR ecosystem)

Sort by relevance (first 500 results collected and screened)

# Test list of benchmark articles

## Bibliographic information of benchmark articles

1. Bizikova, L. and Krcmar, E., 2015. Integrated scenario planning and multi-criteria decision analysis framework with application to forest planning. *Open Journal of Forestry*, *5*(02), p.139.
2. Bremer, L., Mandle, L., Trauernicht, C., Pascua, P., McMillen, H., Burnett, K., Wada, C., Kurashima, N., Quazi, S., Giambelluca, T. and Chock, P., 2018. Bringing multiple values to the table: assessing future land-use and climate change in North Kona, Hawaiʻi. *Ecology and Society*, *23*(1).
3. Burnett, K.M., Ticktin, T., Bremer, L.L., Quazi, S.A., Geslani, C., Wada, C.A., Kurashima, N., Mandle, L., Pascua, P.A., Depraetere, T. and Wolkis, D., 2019. Restoring to the future: Environmental, cultural, and management trade‐offs in historical versus hybrid restoration of a highly modified ecosystem. *Conservation Letters*, *12*(1), p.e12606.
4. Hemmerling, S.A., Barra, M., Bienn, H.C., Baustian, M.M., Jung, H., Meselhe, E., Wang, Y. and White, E., 2019. Elevating local knowledge through participatory modeling: active community engagement in restoration planning in coastal Louisiana. *Journal of Geographical Systems*, pp.1-26.
5. Mitchell, M., Lockwood, M., Moore, S.A. and Clement, S., 2016. Building systems-based scenario narratives for novel biodiversity futures in an agricultural landscape. *Landscape and Urban Planning*, *145*, pp.45-56.
6. Rodorff, V., Steinmetz, L., Mertens, J., Siegmund-Schultze, M. and Köppel, J., 2018. Applying Bayesian networks to evaluate small-scale farmers’ perceptions of native reforestation practices in Brazil’s Caatinga biome. *Regional Environmental Change*, *18*(7), pp.1983-1997.
7. Sisk, T.D., Prather, J.W., Hampton, H.M., Aumack, E.N., Xu, Y. and Dickson, B.G., 2006. Participatory landscape analysis to guide restoration of ponderosa pine ecosystems in the American Southwest. *Landscape and Urban Planning*, *78*(4), pp.300-310.
8. Tobón, W., Urquiza‐Haas, T., Koleff, P., Schröter, M., Ortega‐Álvarez, R., Campo, J., Lindig‐Cisneros, R., Sarukhán, J. and Bonn, A., 2017. Restoration planning to guide Aichi targets in a megadiverse country. *Conservation Biology*, *31*(5), pp.1086-1097.

## Scope of the test list against SPIDER framework

| Article | Sample - Participants | Phenomenon – Ecological Restoration | Design – Scenarios | Evaluation - outcomes | Research type - qualitative, quantitative, mixed methods |
| --- | --- | --- | --- | --- | --- |
| 1 | Industry, business associations, local and regional governments, and non-governmental organizations. | Forest Restoration | 1. Current conditions 2. Diversification of the forest sector | Economic benefits  Total harvest volume  Harvest flows over time | Mixed methods. |
| 2 | Na | Tropical dry ecosystem. | 1. Agroforestry 2. Pasture 3. Coffee 4. Native forest restoration | Cultural services.  Regulating services.  Biodiversity.  Revenue. | Mixed methods. |
| 3 | Conservation managers. | Forest and native species restoration. | 1. Current conditions 2. Historical 3. Hybrid | Ecological  Hydraulic  Cultural | Quantitative |
| 4 | Local residents and resource users. | Coastal restoration | Assessed restoration solutions. | Ecological  Hydraulic | Quantitative |
| 5 | Stakeholder and expert input | Native grassland restoration | NA | Biodiversity | NA |
| 6 | Stakeholders and experts | Native reforestation | NA | Ecosystem health  Smallholder income | Quantitative |
| 7 | NA | Forest restoration | Effects of management decisions. | Fire threat  Wildlife habitat | Quantitative |
| 8 | Stakeholders and experts | National level restoration feasibility. | 11 scenarios | NA | Quantitative |

Table 1. Scope of the test set extracted from the title, abstract and key words against the SPIDER research question design framework. NA refers to components were not indicated.

| Participants: | Experts | General stakeholders | Private sector | Government | Non governmental organisations | Local residents | NA |
| --- | --- | --- | --- | --- | --- | --- | --- |
|  | 4 | 3 | 1 | 1 | 1 | 1 | 2 |
| Ecological Restoration: | Forest restoration | Grassland restoration | Dryland ecosystem | Coastal | Mixed restoration type |  |  |
|  | 4 | 1 | 1 | 1 | 1 |  |  |
| Number of scenarios created: | 1 | 2 | 3 | 4 | 11 | NA |  |
|  | 2 | 1 | 1 | 1 | 1 | 2 |  |
| Number of outcomes examined: | 1 | 2 | 3 | NA |  |  |  |
|  | 1 | 3 | 3 | 1 |  |  |  |
| Research type: | Qualitative | Quantitative | Mixed methods | NA |  |  |  |
|  | 0 | 5 | 2 | 1 |  |  |  |

Table 2. Overall scope of the test list articles.

## Pilot screening of the test list against inclusion and exclusion criteria

| Title and abstract screening | | | |  |
| --- | --- | --- | --- | --- |
| Reference | **Sample: Participants** | **Phenomenon: Ecological Restoration** | **Design: Future Scenarios** | **Evaluation: outcomes** |
| Bizikova et al., 2015 | Yes: *“structured stakeholder groups’ interactions”* | Yes: *“case study of forest restoration”* | Yes: *“modelled scenarios”* | Yes: *“economic benefits, total harvest volumes and harvest flows over time”* |
| Bremer et al., 2018 | Yes: *“native forest restoration”* | Yes: *“participatory deliberative methods”* | Yes: *“land-use scenarios”* | Yes: “*cultural, biodiversity, and ecosystem service value”* |
| Burnett et al., 2019 | Yes: *“hybrid restoration”* | Yes: *“scenarios designed by conservation managers”* | Yes: | Yes: *“ecological, hydraulic and cultural”* |
| Hemmerling et al., 2019 | Yes: *“restoration planning”* | Yes: *“participatory modelling”* | Yes: *“modelling scenarios”* | Yes: *“applicability of solutions”* |
| Mitchell et al., 2016 | Unsure: discusses broadening definition of restoration ecology 🡪 move to full text screening | Yes: *“stakeholder and expert input”* | Yes: *“scenario narratives”* | Yes: *“biodiversity”* |
| Rodorff et al., 2018 | Yes: *“native forest restoration”* | Yes: *“collaboration with stakeholders and experts”* | Yes: *“modelled scenarios”* | Yes: “*conservation of resources and income”* |
| Sisk et al., 2006 | Yes: “*restoration of ponderosa pine”* | Yes: *“participatory landscape analysis”* | Yes: “*modelling alternative management scenarios”* | Yes: “*fire threat and wildlife habitat”* |
| Tobón et al., 2017 | Yes: *“restoration planning”* | Yes: *“engaged stakeholders and experts”* | Yes: *“11 scenarios”* | Yes: “*priority sites”* |

Table 3: Reasoning for inclusion of the test list in title and abstract screening pilot.

| Full text screening | | | | |
| --- | --- | --- | --- | --- |
| Reference | **Sample: Participants** | **Phenomenon: Ecological Restoration** | **Design: Future Scenarios** | **Evaluation: outcomes** |
| Bizikova et al., 2015 | 30 stakeholders included in the process from community, business and government agencies. | Restoration of forests infested with mountain pine beetle. | Uses qualitative scenario planning and then quantified them using multi-criteria decision analysis. | Outcomes focus on socioeconomic and environmental goals e.g. Employment, sales, annual harvest of timber. |
| Bremer et al., 2018 | Scenarios defined through discussions with owners of the land and local community. | Native forest restoration and agroforestry are regarded as restoration scenarios in the paper. | 4 scenarios: current use, native forest restoration, agroforestry, coffee | Net revenue streams for land manager, indigenous cultural and community values, groundwater recharge, fire risk reduction and native biodiversity, |
| Burnett et al., 2019 | Designed research questions with conservation managers, and they described restoration scenarios. | Restoration of an area of a nature preserve comprising of non-native species. | 3 scenarios designed. | Ecological, hydraulic and cultural. |
| Hemmerling et al., 2019 | Technical and local knowledge experts | Costal restoration | 2 participatory scenario models of restoration management. | Land loss, storm surges, salinity intrusion, freshwater intrusion. |
| Mitchell et al., 2016 | Expert input from 27 experts from government, science and rural organisations and land holders. | *Note: although not explicitly clear in the paper*. The goal is to conserve and restore endangered native grassland. | 4 scenarios | Biodiversity outcomes. |
| Rodorff et al., 2018 | Experts provided input into modelling. Model used for quantitative scenarios were build from stakeholders input in a workshop. | Describes the areas as degraded: “free grazing is accountable for the absence of *S. tuberosa* seedlings on degraded Caatinga sites” | Scenarios used for the modelling adoption potential to restore *S tuberosa* in the stud area. | Ecosystem health, agricultural income |
| Sisk et al., 2006 | Targeted experts and stakeholders to guide development and gain data. Did workshops to prioritise restoration areas, management options to then use in scenarios. | Ponderosa pine restoration, particularly due to increasing wildfire risk. | 4 management scenarios created. | Tree stem density, fire hazard, biodiversity density and habitat. |
| Tobón et al., 2017 | Workshop wit 19 experts to design indicators and criteria for scenarios and modelling. Second discussion was used to evaluate scenarios and choose a priority one. | Describes almost half of Mexico as in a degraded state and objective is for priority restoration areas. | 11 scenarios. | Biological importance and restoration feasibility. |

Table 4: Reasoning for inclusion of the test list in full text screening pilot.

## Keyword development for the search string

| Article | Scenario | Ecological | Restoration | Participatory |
| --- | --- | --- | --- | --- |
| 1 | Scenario  Scenario planning  Futures | Species  Forest | Forest Restoration | Stakeholders  Experts |
| 2 | Scenarios | Forest  Ecosystem  Ecological | Forest Restoration | Participatory |
| 3 | Future  Scenarios | Environmental  Ecosystem | Restoring  Restoration  Reforestation | Collaborative |
| 4 | Scenarios | Ecological  Nature-based | Restoration planning  Restoration | Participatory modelling  Engagement  Stakeholders |
| 5 | Scenario  Scenario planning  Biodiversity futures | Ecology | Restoration | Collaborative  Stakeholders |
| 6 | Future  Scenarios | Ecosystem  Environmental | Reforestation | Collaboration |
| 7 | Scenarios | Ecosystem | Restoration  Restore | Participatory  Collaboration  Stakeholder  Collaborative |
| 8 | Scenarios | Ecological  Ecosystem  Biological  Environmental | Restoration  Restoration | Participatory process  Engaged |

Table 5. Keywords present in the title, abstract and keywords of the test set that were relevant to the four main components of the research question.

| Scenario | Ecological | Restoration | Participatory |
| --- | --- | --- | --- |
| Forecasting | Environment | Reforestation | Collaborative |
| Backcasting | Ecosystem | Afforestation | Co-production |
| Future |  | Revegetation | Collective |
| Trajectory |  | Regeneration | Engagement |
|  |  | Afforestation | Stakeholder |
|  |  | Remediation |  |
|  |  | Rehabilitation |  |
|  |  | Rewilding |  |
|  |  | Conservation translocation |  |

Table 6. Key question components and accompanying synonyms taken from the test list, consultation with restoration experts, a librarian, and a thesaurus.

# Screening

## Kappa coefficient for consistency between reviewers

|  | Reviewer 1 initial | Reviewer 2 initial | Kappa |
| --- | --- | --- | --- |
| Title and abstract screening | EM | BP | 0.50 |
|  | EM | SP | 0.36 |
|  | EM | YSF | 0.29 |
| Full text screening | EM | BP | 0.64 |
|  | EM | SP | 0.64 |
|  | EM | YSF | 0.83 |

Table 7. The kappa coefficient calculated between reviewers at each stage of the screening process (1)
